# HIV-related risk behaviours and the correlates among rickshaw pullers of Kamrangirchar, Dhaka, Bangladesh: a cross-sectional study using probability sampling

**DOI:** 10.1186/1471-2458-9-80

**Published:** 2009-03-11

**Authors:** Md Hafiz Ehsanul Hoque, Masako Ono-Kihara, Saman Zamani, Shahrzad Mortazavi Ravari, Masahiro Kihara

**Affiliations:** 1 Department of Global Health and Socio-epidemiology, Kyoto University School of Public Health, Yoshida-Konoe-cho, Sakyo-ku, Kyoto 606-8501, Japan; 2 Directorate General of Health Services, Ministry of Health and Family Welfare, Dhaka, Bangladesh

## Abstract

**Background:**

National HIV serological and behavioural surveillance of Bangladesh repeatedly demonstrated a very high proportion of rickshaw pullers in Dhaka city, having sex with female sex workers (FSWs) and using illicit substances. However, no study has been conducted to identify the correlates of having sex with FSWs among this population. This study aimed to describe behavioural profile of rickshaw pullers in Dhaka city using probability samples and to identify the correlates for having sex with FSWs in order to focus HIV prevention intervention.

**Methods:**

Six hundred rickshaw pullers were randomly selected from rickshaw garages in the Kamrangirchar area, the single largest slum cluster of Dhaka, Bangladesh, during March–April 2008 using the Proportion Probability to Size method. Participants were interviewed, with a response rate of 99.2% (n = 595), using a structured questionnaire and asked about illicit substance use, sexual behaviour and risk perception for HIV and sexually transmitted diseases. Independent predictors of having sex with FSWs were analysed by multivariate analysis. A qualitative study was subsequently conducted with 30 rickshaw pullers to supplement the findings of the initial survey.

**Results:**

The proportion of survey respondents who had sex with FSWs and those who used illicit substances in the previous 12 months period were 7.9% and 24.9%, respectively, much lower than the results achieved in the 2003–04 behavioural surveillance (72.8% and 89.9%, respectively). Multivariate analysis revealed the characteristics of younger age, being never married, living alone with family remaining in other districts and using illicit substances in the previous 12 months were significantly associated with having sex with FSWs.

**Conclusion:**

HIV-related risk behaviour of our study population of the rickshaw pullers was lower than what has been suggested by the results of behavioural surveillance. While this discrepancy should be addressed in further studies, our study emphasizes the importance of focused HIV prevention programs for rickshaw pullers as high-risk behaviour is displayed at an unacceptable level and concentrated in identifiable sub-populations.

## Background

Among the developing countries in Asia, Bangladesh still has a low level HIV epidemic status, where the adult prevalence of HIV infection is estimated to be below 0.1% [1]. However, the overall prevalence of HIV infection among most at-risk populations is increasing with each subsequent round of national HIV serological and behavioural surveillance (from 0.2% in the 2^nd ^round of surveillance to 0.9% in the 7^th ^round of surveillance), mostly due to increased HIV prevalence among injecting drug users (IDUs) [2-7]. According to the results from the recent round of surveillance, the HIV epidemic appears to have reached to a concentrated level (7%) among IDUs in Dhaka city, the capital of Bangladesh [1,2].

In order to explore the future course of the HIV epidemic and to develop the most appropriate prevention programs, it is important to monitor the prevalence of HIV-related risk behaviours among high risk groups, the behaviour networks within and between the high risk groups and their changes over time, which is the role of behavioural surveillance. In 1998, Bangladesh adopted one of the world's most comprehensive behavioural surveillance systems [2]. Updated surveillance has revealed the presence of close sexual networks of IDUs with other high risk groups, especially female sex workers (FSWs) [3,5-7]. FSWs were, on the other hand, shown to have close sexual links with multiple male client groups, not restricted to IDUs. According to recent rounds of behavioural surveillance, rickshaw pullers in Dhaka city are among the client groups of street and brothel based FSWs. The report shows as many as 50% and 72.8% of the rickshaw pullers having sex with FSWs in the last month and 12 months, respectively, mostly without consistently using a condom [5]. As more than 2 million rickshaws are estimated to be operating nationwide [8] and with 0.3 million in Dhaka city (pulled by more than 0.5 million rickshaw pullers) [9], the HIV-related risk behaviours of the rickshaw pullers may have a substantial impact on the future course of the HIV epidemic in Bangladesh. However, one study conducted by Population Council in 6 areas of Dhaka division including 2 areas of Dhaka city demonstrated only 2.69% of married pullers having sex with FSWs in the last 3 months[10]. In addition, in spite of such a potential importance of this population in the context of the HIV epidemic in Bangladesh, there is little intervention activities toward this population; even the correlates of the HIV-related risk behaviours which are critical for a focused intervention program have never been identified. We therefore decided to conduct a cross-sectional study on the HIV-related risk behaviour of rickshaw pullers of Dhaka city in a specified geographical area to accurately describe the HIV-related risk behaviour profile of this population and its correlates using probability samples.

## Methods

### Study design

This study was undertaken using a cross-sectional quantitative design, utilizing structured interviews and complementary qualitative studies before and after the survey. The qualitative investigation undertaken prior to conducting the survey collected information necessary to develop the questionnaire and plan the interview structure. On the other hand, the qualitative investigation after the survey was conducted to supplement and/or reinforce the findings of the survey.

### Study population

The target population for the study was rickshaw pullers renting rickshaws from rickshaw garages, and included rickshaw pullers who kept their own rickshaws in the garages of Kamrangirchar in Dhaka, Bangladesh. This population was targeted because: all of the rickshaw pullers pulled rickshaws inside the city; most of the rickshaw pullers rent carts from the rickshaw garages; most of the garages are concentrated in slum areas; and Kamrangirchar is the single largest slum area bordered with Dhaka city, with approximately 265,000 slum residents [11].

### Sampling procedures

In March 2008, due to the fact that no detailed property-usage map for the Kamrangirchar region was available, the garages in the region were mapped by interviewing local inhabitants and visiting each garage to confirm the location. At the time of the location confirmation, garage information (number of carts for rent and number of rickshaw pullers who rented or kept carts) was collected from the garage owners or managers.

After the list was compiled with information from 213 garages, completeness of the list was further confirmed by three random visits to several locations. Using this list of garages, probability samples of rickshaw pullers were prepared according to the Probability Proportional to Size (PPS) method considering each garage as a cluster [12,13]. Eight pullers were sampled for interview from each cluster. The PPS procedure was used to select 75 garages, in order to obtain the sample size of 600 required for the study, as determined by the method described below. In each chosen garage, a list of the rickshaw pullers who had worked in the previous day was obtained from garage owners or managers and eight rickshaw pullers were randomly selected from the list. Replacement of a selected rickshaw puller was considered when the selected person was not available on the day of the interview.

### Sample size calculation

Considering drug use as a main predictor and having sex with a FSW in the previous 12 months as a main outcome, the sample size was calculated based on previous data on truckers in Dhaka city in which the prevalence of drug use with and without the experience of having sex with FSW in the last 12 months was 67% and 50%, respectively [14]. Assuming expected prevalence of drug use among the rickshaw pullers with and without the experience of having sex with FSW in the previous 12 months to be 70% and 50%, respectively, the sample size required to detect a difference with probability α = 0.05 (two-tailed) and power of 80%, was 206 in total. Taking into consideration the effect of the study design, the expected 88% rate of ever experienced sex among the rickshaw pullers in the last 12 months (extrapolated from the trucker results) and the necessity of adjusting possible confounders, we determined a sample size of 600 was required for the study.

### Interviewing procedures and quality assurance

An explanation of the interview was provided to the owners of the garages selected for participation. Particular efforts were paid to establish good relationship with the owners because the cooperation of them was critical for the success of sampling. The owners were requested to invite the nominated rickshaw pullers to the interviews. In most of the garages, the interviews were carried out early in the morning, according to the owners' preferences. Verbal consent was obtained from each interview participant after an explanation of the nature and purpose of the study. Respondents were also explained about anonymity of the study as their name and address will not be recorded and that they can refuse or stop the interview at any time. The interviews were conducted at a place designated by the participants, in most cases sitting in a rickshaw inside the garage. Fifty taka (US $0.70) was provided for their time for each participant.

The interviews were conducted by nine graduate and undergraduate students from Dhaka University. All interviewers were provided with a one-day training session where they were informed of the study design and purpose and how to conduct the structured interviews. Practice sessions were also provided with discussion of any problems identified during the interviews to determine how the problems should be resolved. One graduate student and the main investigator supervised the interviews to ensure quality was maintained.

### Instrument development

The questionnaire used in the survey was developed on the basis of the Family Health International (FHI) questionnaire [12] and the findings of focus group interviews (FGIs) conducted prior to the survey. Two FGIs with groups of five rickshaw pullers were conducted in a location (Mohammadpur) outside the study area to obtain information on the occupation, lifestyle, sexual behaviour, sensitivity about sexual behaviour queries, and appropriate wording for different behaviours and knowledge. A draft questionnaire was pre-tested on eight rickshaw pullers to verify the wording and face validity. The questionnaire consisted of four fields of questions: demographic characteristics including job experience; illicit substance use; sexual behaviour including condom use; and knowledge and risk perception regarding HIV/sexually transmitted diseases (STDs). The reliability of the instrument was assessed in a test-retest design on 48 independent samples of rickshaw pullers in five Kamrangirchar garages which were not selected for the main survey and with three-day intervals between the tests. Overall, a high test-retest correlation was observed among the continuous variables (Pearson r = 0.86 – 1.00) and among dichotomous variables (Kappa = 0.67 – 1.00).

### Post-survey qualitative study

Immediately after the main survey, in-depth interviews using semi-structured questionnaires were conducted in the streets among 30 rickshaw pullers living in Kamrangirchar and/or renting rickshaws from garages in that area. The purpose of the post-survey qualitative study included gaining detailed information on having sex with FSWs and drug use practices and to evaluate the possible influence of the interview location on the responses to sensitive questions. Interviews were conducted on the street and tape-recorded after obtaining verbal informed consent from the participant. Data were transcribed and analyzed using content analysis.

### Statistical analysis

All statistical analysis was conducted using SPSS Complex Samples 13.0 (SPSS Inc. Chicago, Illinois, USA) to adjust for the effect of clustering. After univariate analysis, bivariate analysis was conducted to assess the association between the experience of having sex with FSWs ('never had sex', 'had sex but not in the last year' and 'had sex in the last year') with predictor variables. Subsequently, multiple logistic regression analysis was performed using a compulsory entry procedure with the presence or absence of having sex with FSWs in the last 12 months as an outcome and other predictor variables that showed crude odds ratios for the outcome with a statistical level of P < 0.1.

### Ethical approval

The study protocol was approved by the Ethical Review committee of Kyoto University, Japan and the Bangladesh Medical Research Council, Dhaka, Bangladesh.

## Results

### Demographic characteristics of the participants

In the study 595 respondents were interviewed, including 50 replacement samples. The response rate was 90.8% for the original samples and 99.2% when replacements were included. Only two rickshaw pullers from the original list of interview candidates and none of the replacement samples refused to participate in the interview. As shown in Table 1, 90.6% of rickshaw pullers were from districts outside Dhaka city. The mean age of the participants was 32.1 years (SE 0.40) and 89.6% were or had been married. Among the married participants, though not shown in Table 1, 13.7% had been married more than once. In terms of formal education, 62.5% of the participants did not attend formal schooling and only 14.8% completed primary school (Class I to class V). Among the participants 45.4% periodically came to Dhaka to work and 42.5% were living alone in Dhaka, apart from their families. The average time spent in the rickshaw pulling profession was 8.6 years and the mean income of the respondents in the previous month was 6332 taka (~USD 90). Of the participants, 99.0% nominated Muslim as their religion.

**Table 1:** Sociodemographic characteristics of rickshaw pullers in Kamrangirchar area of Dhaka, Bangladesh (n = 595)

	n	% of total	95% CI
Age			
≤ 20 years	39	6.6	4.6 – 9.3
21 to 30 years	296	49.7	45.7 – 53.8
31 to 40 years	153	25.7	22.4 – 29.4
41 to 50 years	85	14.3	11.7 – 17.4
> 50 years	22	3.7	2.5 – 5.4
Mean age (years)	32.12 (SE 0.40)		31.33 – 32.91
Marital status			
Ever married	533	89.6	86.6 – 91.9
Never married	62	10.4	8.1 – 13.4
Education			
No schooling	372	62.5	58.3 – 66.5
Up to primary school	135	22.7	19.2 – 26.6
Up to secondary school	81	13.6	11.0 – 16.7
More than secondary school	7	1.2	0.5 – 2.7
Mean years in education	2.03 (SE 0.13)		1.78 – 2.29
Religion			
Islam	589	99.0	97.2 – 99.6
Hinduism	6	1.0	0.4 – 2.8
Living arrangement			
With family	337	56.6	49.1 – 63.8
Alone, family apart	253	42.5	35.3 – 50.1
No family	5	0.8	0.4 – 2.0
Home district			
Other than Dhaka	539	90.6	86.9 – 93.3
Dhaka	56	9.4	6.7 – 13.1
Periodical to Dhaka			
Yes	270	45.4	38.4 – 52.5
No	325	54.6	47.5 – 61.6
Mean years in profession	8.64 (SE 0.33)		7.99 – 9.30
Mean income in last month (Taka)	6332 (SE 97)		6138 – 6527

CI, confidence interval incorporating cluster effect

SE, standard error

### Sexual behaviour and related events

Table 2 illustrates the sexual behaviour of the participants. Most of the participants had experienced sex (95.8%), with the average age at sexual debut being 19.8 years. With regard to the type of sexual partners ever, 91.9% had experienced sex with regular female partners, 30.1% with FSWs and 19.8% with casual female partners.

**Table 2:** Sexual behaviour profile and related events among rickshaw pullers of Kamrangirchar area of Dhaka, Bangladesh (n = 595)

Sexual experience		n	% of total	95% CI
Ever had sex		570	95.8	93.9 – 97.1
Had sex in the last 12 months		550	92.4	89.8 – 94.4
Age of first sex (mean)		19.78 (SE 0.16)		19.45 – 20.10
Sex with regular partners				
Ever had it		547	91.9	89.4 – 93.9
Had it in the last 12 months		531	89.2	86.3 – 91.6
Condom use in last 12 months (n = 531)	Every time	11	2.1	1.1 – 3.8
	Sometime	93	17.5	14.2 – 21.4
	Never	427	80.4	76.3 – 84.0
Used condom at last sex (n = 531)		33	6.2	4.3 – 8.8
Sex with casual partners				
Ever had it		118	19.8	17.0 – 23.0
Had it in the last 12 months		8	1.3	0.6 – 2.8
Condom use in the last 12 months (n = 8)	Every time	2	25.0	7.5 – 57.9
	Sometimes	0	0.0	
	Never	6	75.0	42.1 – 92.5
Used condom at last sex (n = 8)		2	25.0	7.5 – 57.9
Sex with female sex workers (FSW)				
Ever had it		179	30.1	26.1 – 34.4
Had it in the last 12 months		47	7.9	6.0 – 10.4
Had it in the last month		27	4.5	3.2 – 6.4
Condom use in the last 12 months (n = 47)	Every time	14	29.8	18.2 – 44.7
	Sometime	15	31.9	20.6 – 45.9
	Never	18	38.3	25.3 – 53.2
Used condom at last sex (n = 47)		19	40.4	26.5 – 56.1
Group sex with female sex workers				
Ever had it		28	4.7	3.1 – 7.0
Had it in the last 12 months		7	1.2	0.6 – 2.4
Condom use in the last 12 months (n = 7)	Every time	3	42.9	13.9 – 77.6
	Sometimes	2	28.6	6.9 – 68.2
	Never	2	28.6	6.9 – 68.2
Used condom at last sex (n = 7)		4	57.1	22.4 – 86.1
Sex with male partners				
Ever had it		13	2.2	1.3 – 3.8
Had it in the last 12 months		5	0.8	0.4 – 2.0
Condom use in the last 12 months (n = 5)	Every time	2	40.0	9.6 – 80.6
	Sometimes	1	20.0	2.6 – 70.2
	Never	2	40.0	9.6 – 80.6
Used condom at last sex (n = 5)		2	40.0	9.6 – 80.6
Number of sex partners in the last 12 months among who ever had sex (n = 570)				
All partners	0	20	3.5	2.3 – 5.4
	1	490	86.0	83.2 – 88.3
	2 to 4	28	4.9	3.4 – 7.0
	≥ 5	32	5.6	4.0 – 7.8
Female sex workers	0	523	91.8	89.1 – 93.8
	1	3	0.5	0.2 – 1.6
	2 to 4	15	2.6	1.6 – 4.3
	≥ 5	29	5.1	3.6 – 7.2
Ever diagnosed with sexually transmitted diseases		73	12.3	9.6 – 15.6
Ever tested for HIV		9	1.5	0.7 – 3.0
Exposure to prevention services		46	7.7	5.9 – 10.0

CI, confidence interval incorporating cluster effect; SE, standard error

Denominators (n) are shown in parentheses for condom use and the number of sex partners

Among the survey participants, 92.4% of the respondents were sexually active in the last year. With regard to the type of sexual partners in the last year, 89.2% of the respondents reported having sex with regular female partners, while only 1.3% had sex with casual female partners, 7.9% with FSWs and 0.8% with male sex partners. Among the participants who had sex with FSWs, seven people reported to having shared a single FSW with other male clients in the last year (group sex). Sexually active respondents reported that in the previous year, 10.5% have had more than one sex partner and 5.6% had five or more partners for all sex partners, whereas it was 7.7% and 5.1% respectively for FSW partners.

Consistent condom use was very low (2.1%) for those with regular female partners, low (25–30%) with FSWs and casual female partners and moderate (≥ 40%) in group sex or with male sex partners. The frequency of condom use in the last sexual experience was similar to or approximately 10–15% higher than the frequency of consistent condom use in the previous 12 months. Though not shown in the table, among the sexually experienced respondents, 96.1% (95% CI, 94.2 – 97.4) reported to having had vaginal sex in the previous year, 4.4% (95% CI, 3.0 – 6.5) oral sex, and 3.2% (95% CI, 2.0 – 4.9) anal sex.

Among all respondents, 12.3% reported to having ever suffered from STDs, while only 1.5% had ever been tested for HIV and only 7.7% exposed to AIDS/STI prevention services by any public agency or Non Government Organisation (NGO).

### Illicit substance use

Of all respondents 45.7% (95% CI, 40.6 – 50.9) reported to having used illicit substances in their lifetime and 24.9% (95% CI, 20.3 – 30.1) had used them in the last 12 months. Cannabis was most common (94.6%; 95% CI, 89.4 – 97.3) illicit substance used by the respondents. Among the users, three quarters (73.5%, 95% CI 65.5 – 80.1) used illicit substances with a frequency of several days a week or more in the previous 12 months. No respondent claimed to have injected drugs.

### Knowledge and risk perception

Among all participants, 92.8% (95% CI, 90.5 – 94.5) reported to having heard the word "AIDS", 33.1% (95% CI, 28.4 – 38.1) believed that AIDS can be cured by treatment, and only 57% (95% CI, 52.9 – 61.0) believed that using condom could provide protection from getting AIDS. Regarding AIDS risk perception, only 4.5% (95% CI, 3.1 – 6.5) of respondents considered themselves at risk of getting the infection whereas 68.1% (63.0 – 72.7) considered themselves at no risk.

### Correlates of having sex with FSWs

Table 3 compares demographic or behavioural characteristics among three subgroups of the pullers having different sexual experience with FSWs: never had sex with FSWs; had sex with FSWs but not in the last 12 months; and had sex with FSWs in the last 12 months. In Table 3, data are shown in two rows for each variable where the upper row represents the prevalence of the answer indicated in the upper row (e.g. Age ≤ 30 years) among each subgroups while the lower row represents odds ratio and 95% confidence interval referred to the category shown in the parenthesis (eg. Aged > 30 years) with the subgroup of "Never had sex with FSWs" as a baseline. Most of the variables showed an association in terms of crude odds ratio becoming stronger or strongest with the category of "Had sex with FSWs in the last 12 months" except for schooling and income in the last month. Based on these analyses, "Never had sex with FSWs" and "Had sex with FSWs but not in the last 12 months" were merged to create a dichotomous outcome variable representing the presence or absence of having sexual intercourse with FSWs in the last 12 months.

**Table 3:** Bivariate association of demographic and behavioural variables with having sex with female sex workers (FSWs) among sexually active rickshaw pullers in Kamrangirchar area of Dhaka, Bangladesh (n = 570)

	% of referred answer, odds ratios and 95% CI		
	
	Never had sex with FSWs (n = 391)	Ever had sex with FSWs but not in the last 12 months (n = 132)	Had sex with FSWs in the last 12 months (n = 47)
**Demographic variables**			
Aged ≤ 30 years	51.9	52.3	80.9
(referred to >30 years)	1	1.01(0.68 – 1.51)	3.91(1.84 – 8.30)
Never married	2.8	9.1	29.8
(referred to married)	1	3.46 (1.31 – 9.14)	14.66 (6.35 – 33.84)
No schooling	65.0	63.6	63.8
(referred to with schooling)	1	0.94 (0.61 – 1.47)	0.95 (0.48 – 1.87)
Living alone	38.9	40.2	72.3
(referred to living with family)	1	1.06 (0.72 – 1.55)	4.11 (2.09 – 8.09)
Came from other district	90.8	87.1	97.9
(referred to from Dhaka)	1	0.69 (0.35 – 1.35)	4.67(0.57 – 38.27)
Coming periodically to Dhaka	43.2	42.4	66.0
(referred to not coming periodically)	1	0.97 (0.63 – 1.49)	2.55 (1.39 – 4.66)
Year in profession ≤ 6 years	44.8	38.6	61.7
(referred to > 6 years)	1	0.78 (0.50 – 1.21)	1.99 (1.04 – 3.80)
Income in last month > 6000 taka	52.4	53.0	53.2
(referred to ≤ 6000 taka)	1	1.02 (0.70 – 1.50)	1.03 (0.54 – 1.97)
**Behavioural variables**			
Used substance in last 12 months	16.4	40.2	61.7
(referred to did not use)	1	3.43 (2.14 – 5.48)	8.23 (4.27 – 15.89)
Hadn't sex with wife in last 12 months	1.1	0.8	6.1
(referred to had)	1	0.79 (0.08 – 7.41)	6.07 (1.21 – 30.36)
Had sex with casual partner in last 12 months	1.0	0.8	6.4
(referred to did not have)	1	0.74 (0.08 – 7.13)	6.60 (1.03 – 42.46)
Had sex with male in the last 12 months	0.3		8.5
(referred to did not have)	1		36.28 (3.88 – 339.14)
Had oral sex in the last 12 months	1.3	8.3	21.7
(referred to did not have)	1	6.78 (2.17 – 21.12)	20.89 (6.65 – 65.59)
Had anal sex in the last 12 months	0.8	3.3	23.9
(referred to did not have)	1	4.31(0.91–20.32)	39.60 (9.71 – 161.53)
Ever suffered from STDs	7.7	20.5	34.0
(referred to never/do not know)	1	3.09 (1.69 – 5.67)	6.21 (2.96 – 13.03)
Perceived risk for HIV infection	1.3	6.8	25.5
(referred to did not perceive/don't know)	1	5.65 (1.78 – 17.95)	26.47 (9.29 – 75.39)
Ever exposed to intervention	6.9	8.3	17.0
(referred to never)	1	1.23 (0.56 – 2.69)	2.77 (1.18 – 6.50)

CI, confidence interval

STDs, sexually transmitted diseases

Table 4 illustrates the results of bivariate and multivariate analyses using the dichotomous outcome variable. In the multivariate analysis, all variables were compulsorily entered except for the variables which do not correlate with the outcome at a significance level of *P *< 0.1 by bivariate analyses. Variables that showed extremely biased response distribution and the variable of "periodical pulling" that are highly (r > 0.8) interrelated with the variable of "living alone" were excluded. Multivariate analysis showed that participants who are aged 30 years or below, never married, living alone, used illicit substances in the last 12 months, or had oral or anal sex in the last 12 months were significantly more likely to have had sex in the previous 12 months with FSWs, while the number of years in the profession, experience of STD in lifetime and the exposure to HIV prevention services did not show significant association. The association with the outcome was strong (adjusted odds ratio [AOR]>10) for marital status and anal sex, moderate (AOR>5) for oral sex and perceived risk of HIV infection, and mild (AOR>3) for living arrangement and illicit substance use.

**Table 4:** Bivariate and multivariate association of demographic and behavioural variables with having sex with female sex workers (FSWs) among sexually active rickshaw pullers in Kamrangirchar area of Dhaka, Bangladesh

	Bivariate analysis (n = 570)							Multivariate analysis (n = 548)		
		
			% distribution of answers in subpopulations							
									
	n	% of total	Haven't had sex with FSWs in the last 12 months	Had sex with FSWs in last the 12 months	Crude odds ratio	95%CI	*P*	Adjusted odds ratio	95%CI	*P*
Age group										
≤30 years	310	54.4	52.0	80.9	3.90	1.88 – 8.07	0.000	2.46	1.03 – 5.91	0.044
> 30 years (ref)	260	45.6	48.0	19.1						
Ever married										
Never married	37	6.5	4.4	29.8	9.22	4.40 – 19.35	0.000	11.44	2.97–44.00	0.001
Married (ref)	533	93.5	95.6	70.2						
Schooling										
No schooling	368	64.6	64.6	63.8	0.97	0.50 – 1.87	0.917			
Schooling (ref)	202	35.4	35.4	36.2						
Living arrangement										
Alone	239	41.9	39.2	72.3	4.06	2.09 – 7.88	0.000	3.11	1.45 – 6.66	0.004
With family (ref)	331	58.1	60.8	27.7						
Home district										
Other districts	516	90.5	89.9	97.9	5.19	0.64 – 42.21	0.122			
Dhaka (ref)	54	9.5	10.1	2.1						
Periodical pulling										
Yes	256	44.9	43.0	66.0	2.57	1.42 – 4.65	0.002			
No (ref)	314	55.1	57.0	34.0						
Years in profession										
≤ 6 years	255	44.7	43.2	61.7	2.12	1.10 – 4.06	0.025	0.99	0.42 – 2.36	0.983
> 6 years (ref)	315	55.3	56.8	38.3						
Monthly income										
> 6000 taka	300	52.6	52.6	53.2	1.03	0.53 – 1.98	0.941			
≤ 6000 taka (ref)	270	47.4	47.4	46.8						
Substance used in last 12 months										
Yes	146	25.6	22.4	61.7	5.59	2.98 – 10.51	0.000	3.12	1.30 – 7.52	0.012
No (ref)	424	74.4	77.6	38.3						
Had sex with wife in last 12 months										
No	7	1.3	1.0	6.1	6.39	1.36 – 30.11	0.020			
Yes (ref)	526	98.7	99.0	93.9						
Had sex with NRNC partner in last 12 months										
Yes	8	1.4	1.0	6.4	7.06	1.17 – 42.84	0.034			
No (ref)	562	98.6	99.0	93.6						
Had group sex in last 12 months										
Yes	7	1.2	0.0	14.9						
No (ref)	563	98.8	100.0	85.1	NA					
Had sex with male in last 12 months										
Yes	5	0.9	0.2	8.5						
No (ref)	565	99.1	99.8	91.5	48.56	5.17–456.49	0.001			
Oral sex										
Yes	25	4.6	3.0	21.7	9.02	3.65 – 22.28	0.000	5.06	1.58–16.23	0.007
No (ref)	523	95.4	97.0	78.3						
Anal sex										
Yes	18	3.3	1.4	23.9	22.22	7.48 – 66.00	0.000	18.75	4.44–79.14	0.000
No (ref)	530	96.7	98.6	76.1						
Ever experienced STD										
Yes	73	12.8	10.9	34.0	4.22	2.14 – 8.34	0.000	2.52	0.88–7.21	0.084
No/do not know (ref)	497	87.2	89.1	66.0						
Perceived risk of HIV infection										
At risk	26	4.6	2.7	25.5	12.47	5.74 – 27.07	0.000	7.26	1.79–29.43	0.006
Not at risk/do not know (ref)	544	95.4	97.3	74.5						
Exposure to prevention services										
Yes	46	8.1	7.3	17.0	2.62	1.17 – 5.85	0.020	1.81	0.55–5.94	0.324
No (ref)	524	91.9	92.7	83.0						

95% CI, 95% confidence interval; ref, reference category in the calculation of odds ratio

### Results of the post survey qualitative study

Content analysis of the in-depth interview data from the 30 rickshaw pullers showed similar demographics and behavioural profiles to the main survey. On average, more than a third (40%) of participants had sex with FSWs in their lifetime but only 3.3% did so in the previous 12 months. Illicit substance use in the previous 12 months was reported by only a third (33.3%) of the participants.

## Discussion

This is the first study applying the PPS method to the survey of rickshaw pullers in Dhaka, Bangladesh, in an attempt to describe more accurate profile of this population in regards with HIV-related risk behaviours and the correlates. Our findings, in contrast to what was previously reported by the HIV surveillance program in Dhaka, showed much lower proportion of rickshaw pullers reported having sex with FSWs in the previous 12 months. Multivariate analyses also showed that the characteristics of younger age, being never married, living alone and using illicit substances in the previous 12 months were significantly associated with having sex with FSWs in the previous 12 months.

Since 2000, the rickshaw pullers of Dhaka city have been one of the sentinel groups of the national HIV serological and behavioural surveillance survey (BSS) and have been classified as one of the important bridging populations for HIV infection. Data from behavioural surveillance in 2003-04 showed that 90% of the rickshaw pullers were identified as using illicit substances (Cannabis) and more than 70% as having sex with FSW in the previous 12 months.

However, the profile of HIV-related risk behaviour of our study population is very different from that of the surveillance program in Bangladesh. In our survey, the frequency of having sex with FSWs and illicit substance use in the previous 12 months was only 7.9% and 24.9%, respectively, both results much lower than those reported in the surveillance (72.8% and 89.9%). Frequency of sex with FSWs of our study population appeared to be close to that of general male population. The proportion of the pullers who ever had sex with FSWs in our study (30.1%) was almost equivalent to that (32%) of the married male population as observed in a randomized survey of the Health and Demographic surveillance in Bangladesh conducted in 2004 in 2 rural areas of southern part of Bangladesh[15]. Not only sexual behaviour, differences also existed in the demographic profiles between our study and the surveillance. Compared to the 2003–04 BSS data our samples were older (32.1 vs. 28.4 years), in the profession longer (8.6 vs. 6.1 years), more likely to have been married (89.6 vs. 76.5%) and more lacking formal education (62.5 vs. 33.3%).Reasons for such a discrepancy between our study and the surveillance could include the difference in geographical area covered by each study, the difference in the year when the studies were conducted, difference in methodologies such as sampling methods, the difference in the places where the interviews were conducted, and the possible selection bias introduced in the process of sampling.

Regarding the geographical coverage, our study population, though all operate in the city, represents only Kamrangirchar area, while the surveillance covers entire Dhaka city. It may be that the pullers residing in Kamrangirchar may be different in demographic and behavioural profiles from the rest of the pullers operating in the city area. But similar demographic and behavioural profiles of the pullers in Dhaka city were reported from 2 other recent studies. One is the survey by the Population Council conducted in 2004–05 as a baseline survey (n = 973) of community intervention study for HIV prevention. The other is the survey by Rahman et al in 2003 (n = 1000) to investigate the smoking behaviour of the pullers[16]. The former adopted a garage-based sampling in the six different high risk locations of Dhaka division including two locations within Dhaka city other than Kamrangirchar. The later study recruited the pullers from all 10 blocks of the Dhaka city by approaching from four different corners of each block to the centre recruiting one puller in each waiting spot along the way. Population Council survey indicated only 2.69% of married pullers reported having sex with FSWs during the last 3 months among all respondents, similar to our results (4.5% in the last one months). Participants of these studies were older (35 and 32.5 years, respectively) and more lacking formal education (62 and 60%, respectively), also consistent with the results of our survey. Interpretation of these studies however requires some caution because response rate was low in the Population Council survey (45%) and because samples were not selected in an equal probability in Rahman's study. More studies are clearly needed to accurately describe the characteristics of the entire population of the pullers in Dhaka city.

Difference in the year of survey could be another reason for the difference since our survey was conducted in 2008, more than 3 years later the last surveillance (fifth round in 2003–04). It may be that radical changes in both demographic and behavioural profiles of the pullers operating in the city area may have occurred during such a short period of time. But this is difficult to assume because Rahman's survey which reported the similar demographic profiles of the pullers to our survey, was conducted in 2003. Moreover, there was little intervention among this group as only 1.5% of our participants were ever tested for HIV and only 7.7% were ever exposed to prevention services by government or NGO health worker.

Difference in the methodologies employed in each survey could be the cause of discrepancy. While our survey used the list of pullers registered at a garage as a cluster for the second stage random sampling, group of pullers at a waiting spot that is more mobile in number or membership over time was used in the surveillance. Such a difference in the nature of sampling frame may have somehow influenced the characteristics of the samples.

Difference in the places for interview might have influenced the pattern of responses. Our interview was conducted mostly inside garage but it was on the street in the surveillance. Because of the lower anonymity of garage environment compared to the street, participants of our survey might have responded in a socially desirable way in terms of sexual activities or substance use. This is however unlikely because results of content analysis of our qualitative study conducted immediately after the survey among 30 pullers of Kamrangirchar recruited on the street were almost identical to the survey results.

Finally, difference could be due to the bias introduced in the process of sampling. Though we could achieve high response rate over 90%, response rate has not been described in the last round of behavioural surveillance (2003–04) report of Bangladesh. If the response rate in the surveillance was low, it is possible that pullers who were younger and more open to talk about sexual experience or substance use, responded selectively to the interview more than those who were not.

Though our study population showed much less HIV-related risk behaviour compared to the surveillance, there is no room for complacency in view of the context of the HIV epidemic in Bangladesh. Of the rickshaw pullers who had sex with FSWs in the last 12 months, more than 70% did not consistently used condoms. Two thirds of those rickshaw pullers were married and rarely (2.1%) used condoms with their regular partners. It is therefore possible that with the advancing HIV epidemic among IDUs and FSWs in the future, rickshaw pullers may be involved in the epidemic and bridge the epidemic into the general population.

It is also important to note that one quarter of the participants knew little about HIV/AIDS and in our survey only 7.7% of the participants reported having ever been exposed to HIV/AIDS intervention by the government or NGO health workers. If this situation remains unchanged, it would be difficult for rickshaw pullers to comprehend the real risk of HIV infection and to take preventive actions. As the HIV infection is yet to be concentrated among the FSW, there is still opportunity for Bangladesh to prevent the HIV epidemic among rickshaw pullers by providing correct knowledge and information.

The factors correlated with having sex with FSWs identified in our survey may be helpful in this respect. It was shown that rickshaw pullers who never married, those who were younger and those who were living apart from family were likely to buy sex. Though small in number, pullers who never married (n = 37) are of particular concern since 37.8% compared to 6.2% of married pullers reported sex with FSWs in the previous 12 months (calculated from Table 3). Younger pullers (≤ 30 years) or those living apart from families are also important because they represent around half of the entire puller population and 12–15% of them reportedly had sex with FSWs in the previous 12 months. Prevention programs toward rickshaw pullers could be multifaceted including the use of opinion leaders or peer counselling. Garage may also be an excellent place for intervention. These programs however should include a clear focus on unmarried and younger subpopulations and particularly those living apart from families to prevent the possible bridging of the HIV infection to their families. In view of the strong association of oral and anal sex with having sex with FSWs, it is important to inform rickshaw pullers who participating in anal sex about the risk of direct HIV infection; and the pullers who participating in oral sex about the increased risk of contracting STDs that indirectly enhances the risk of HIV infection.

Our study has limitations. We conducted the survey with rickshaw pullers in the Kamrangirchar area which may not represent the entire population of rickshaw pullers in Dhaka city. Sampling only considered rickshaw pullers who pulled rickshaws on the previous day, which may have reduced the number of rickshaw pullers in the sampling framework.

## Conclusion

Our study, using probability samples of the rickshaw pullers by PPS method portrayed a different HIV-related risk behaviour profile from that had been suggested by the national BSS in Bangladesh. Our results suggest that a segment of rickshaw pullers are at risk for HIV infection and there is still opportunity of preventing HIV epidemic among them and their family by swiftly crafting and expanding the prevention program.

## Competing interests

The authors declare that they have no competing interests.

## Authors' contributions

All authors contributed to the design of this study. EH conduct the field survey and performed analysis. MOK design the qualitative part of the study including designing the procedure for qualitative survey and guided overall study procedure including revised manuscript. MK guided overall study procedure including analyzing data and drafting and revising manuscript. SZ designed statistical procedures in the field and statistical analysis including revision of manuscript. SMR performed analysis of qualitative data. All authors read and approved the final manuscript.

## Pre-publication history

The pre-publication history for this paper can be accessed here:



## References

[B1] UNAIDS (2008). Report on global AIDS epidemic: 2008.

[B2] HIV surveillance in bangladesh. http://www.icddrb.org/images/HIV_surveillance_in_Bangladesh.pdf.

[B3] Chan Philip A, Khan Omar A (2007). Risk factors for HIV infection in Males who have Sex with Males (MSM) in Bangladesh. BMC Public Health.

[B4] (2004). National HIV Serological Surveillance, Bangladesh: Sixth Round Technical Report.

[B5] (2003). National HIV Serological and Behavioural Surveillance, Bangladesh: Fifth Round Technical Report.

[B6] (2002). National HIV Serological and Behavioural Surveillance, Bangladesh: Fourth Round Technical Report.

[B7] (2001). National HIV Serological and Behavioural Surveillance, 2000-Bangladesh: Third Round Report.

[B8] (2006). Statistical Pocket Book of Bangladesh.

[B9] (2006).

[B10] Bhuiya I, Rahman M, Rob U, Khan ME, Zahiduzzaman (2007). Increasing Dual Protection among Rickshaw Pullers in Bangladesh.

[B11] Centre for Urban Studies (CUS), National Institute of Population Research and Training (NIPORT), Measure Evaluation (2005). Slums in Urban Bangladesh: Mapping and Census. Dhaka.

[B12] (2000). Behavioral Surveillance Surveys: Guide lines for repeated Behavioral Surveys in Populations at Risk of HIV.

[B13] Nishimura Yumiko H, Ono-Kihara Masako, Mohith Jagdis C, NgManSun Renaud, Homma Takayuki, DiClemete Ralph J, Lang Delia L, Kihara Masahiro (2007). Sexual behaviors and their correlates among young people in Mauritius: a cross-sectional study. BMC International Health and Human Rights.

[B14] Gibney Laura, Saquib Nazmus, Metzger Jesse (2003). Behavioral risk factors for STD/HIV transmission in Bangladesh's trucking industry. Social Science & Medicine.

[B15] Mercer Alex KR, Gurley Emily, Azim Tasnim (2007). Sexual Risk Behavior of Married Men and Women in Bangladesh Associated With Husbands' Work Migration and Living Apart. Sexually Transmitted Diseases.

[B16] Rahman M, Awal ASMN, Fukui T, Sakamoto J (2007). Prevalence of Cigarette and Bidi Smoking among Rickshaw Pullers in Dhaka City. Preventive Medicine.

